# *Galnt1* Is Required for Normal Heart Valve Development and Cardiac Function

**DOI:** 10.1371/journal.pone.0115861

**Published:** 2015-01-23

**Authors:** E Tian, Sharon R. Stevens, Yu Guan, Danielle A. Springer, Stasia A. Anderson, Matthew F. Starost, Vyomesh Patel, Kelly G. Ten Hagen, Lawrence A. Tabak

**Affiliations:** 1 Developmental Glycobiology Section, Laboratory of Cell and Developmental Biology, National Institute of Dental and Craniofacial Research, National Institutes of Health, Bethesda, United States of America; 2 Section on Biological Chemistry, National Institute of Dental and Craniofacial Research, National Institutes of Health, Bethesda, United States of America; 3 National Heart, Lung, and Blood Institute, National Institutes of Health, Bethesda, United States of America; 4 Division of Veterinary Resources, National Institutes of Health, Bethesda, United States of America; 5 Oral and Pharyngeal Cancer Branch, National Institute of Dental and Craniofacial Research, National Institutes of Health, Bethesda, United States of America; Cincinnati Children’s Medical Center, UNITED STATES

## Abstract

Congenital heart valve defects in humans occur in approximately 2% of live births and are a major source of compromised cardiac function. In this study we demonstrate that normal heart valve development and cardiac function are dependent upon *Galnt1*, the gene that encodes a member of the family of glycosyltransferases (GalNAc-Ts) responsible for the initiation of mucin-type O-glycosylation. In the adult mouse, compromised cardiac function that mimics human congenital heart disease, including aortic and pulmonary valve stenosis and regurgitation; altered ejection fraction; and cardiac dilation, was observed in *Galnt1* null animals. The underlying phenotype is aberrant valve formation caused by increased cell proliferation within the outflow tract cushion of developing hearts, which is first detected at developmental stage E11.5. Developing valves from *Galnt1* deficient animals displayed reduced levels of the proteases ADAMTS1 and ADAMTS5, decreased cleavage of the proteoglycan versican and increased levels of other extracellular matrix proteins. We also observed increased BMP and MAPK signaling. Taken together, the ablation of *Galnt1* appears to disrupt the formation/remodeling of the extracellular matrix and alters conserved signaling pathways that regulate cell proliferation. Our study provides insight into the role of this conserved protein modification in cardiac valve development and may represent a new model for idiopathic valve disease.

## Introduction

Congenital heart valve defects in humans have been estimated to occur in approximately 2% of live births and valve defects of some type occur in about 30% of cardiovascular malformations [1]. Despite this high incidence, the genetic basis for valvular defects remains incompletely defined, although clearly integrated regulatory networks including signal transduction pathways [2, 3, 4], coupled to programmed changes in the extracellular matrix (ECM) [5, 6], underlie the normal formation of these key structures. Indeed, ECM synthesis and remodeling play important roles in regulating the migration and proliferation of cells that will eventually give rise to the heart valves later in development [6]. Valve developmental mechanisms are conserved among vertebrate species including human, mouse and chicken, although morphological and structural differences exist [7].

Recent work demonstrated an essential role played by the protein modification, termed “mucin-type” O-glycosylation, in influencing the composition of the ECM during development [8]. Mucin-type O-glycosylation is initiated by the family of enzymes known as the UDP-GalNAc:polypeptide α-*N*-acetylgalactosaminyltransferases (GalNAc-Ts, EC 2.4.1.41). These glycosyltransferases catalyze the transfer of *N*-acetylgalactosamine (GalNAc) to the hydroxyamino acids, threonine (Thr) and serine (Ser), of proteins destined to be membrane-bound or secreted [9]. The ECM surrounding the developing salivary glands in mice lacking a member of this family (*Galnt1*) was significantly altered, resulting in decreased levels of both laminin and collagen IV, diminished FGF-mediated signaling and decreased cell proliferation [8]. *Galnt1* nulls also exhibit bleeding disorders, impaired leukocyte trafficking and reduced IgG production [10]. Here we demonstrate that *Galnt1* nulls also present with cardiovascular abnormalities, the result of aberrant embryonic heart valve development. Interestingly, loss of *Galnt1* in the developing heart results in altered abundance of proteases, ECM proteins and changes in signaling pathways associated with increased cell proliferation. Our studies define a role for *Galnt1* in embryonic heart valve development and in subsequent cardiac function in adults.

## Materials and Methods

### Animal Breeding and Genotyping

The generation of *Galnt1* (previously designated ppGalNAcT-1) deficient mice has been described previously [10]. *Galnt1-*deficient mice were backcrossed into the C57BL/6NHsd inbred mouse background for over 10 generations before analysis. Heterozygous *Galnt1* animals (*+/-*) were crossed to generate embryos of the genotypes denoted. Embryos were genotyped by PCR according to standard procedures and as described previously [10]. Wild type (*Galnt1^+/+^*) and homozygous *Galnt1-*deficient (*Galnt1^-/-^*) siblings were compared in all experiments. Experimental procedures were reviewed and approved by the Animal Care and Use Committee of the National Institutes of Health (ASP protocol #11–596).

### Histological Analysis

Mouse hearts or embryos were fixed in 4% PFA/PBS at room temperature overnight, then transferred to 70% ethanol and stored at 4°C. Paraffin-embedded tissue sections (5 μm) were used for hematoxylin/eosin (H&E) staining.

### 2D and M-mode Echocardiograph

Transthoracic echocardiography was performed with a high frequency linear array ultrasound system (VisualSonics, Vevo 2100). All images were acquired using a MS-400 transducer (VisualSonics) with a center operating frequency of 30MHz, and broadband frequency of 18–38MHz. The axial resolution of this transducer is 50um, and the footprint is 20mmx5mm. M-mode images of the left ventricle were collected from the parasternal short axis view at the level of the mid papillary muscles; obtained by rotating the probe 90 degrees clockwise from the parasternal long axis view. From the M-mode images the left ventricle (LV) systolic and diastolic posterior and anterior wall thicknesses, as well as end systolic and end diastolic internal LV chamber dimensions (LVIDs, LVIDd) were measured using the leading edge method. LV functional values of fractional shortening (FS) and ejection fraction (EF) were calculated from the wall thicknesses and chamber dimension measurements through system software as: FS % = 100 ((LVID;d-LVIS;s)/LVID;d), %EF = 100 ((LV vol;d-LV vol;s)/LV Vol;d) where LV vol;d = ((7.0/(2.4 + LVID;d)) x LVID;d^3^ and LV Vol;s = ((7.0 / (2.4 x LVID;s)) x LVID;s^3^.

### Pulsed-Wave (PW) and Color Doppler

Color Doppler ultrasonography was also performed using the high frequency ultrasound system (VisualSonics, Vevo 2100) equipped with the MS-400 30 MHz linear array transducer that generated a spectral Doppler frequency of 24 MHz. Color flow profiles were obtained for the left ventricular outflow tract (LVOT) and ascending aorta from a suprasternal view. For generating the pulmonary artery color flow profiles, the probe was moved slightly cranial and leftward from the parasternal long axis view until the pulmonary artery was observed crossing over the aorta. The color flow images served as a guide for placement of the PW Doppler sample volume in order to obtain peak aortic and pulmonary artery velocities. The angle of insonation was < 15 degrees for pulmonary artery (PA) measurements, and < 45 degrees for LVOT/ascending aorta. The mice were lightly anesthetized with 0.5–1.0% Isoflurane to achieve a target heart rate of > 400 beats per minute, and were positioned over a heated platform during all imaging studies.

### Immunofluorescence

Fixed, paraffin-embedded tissue sections (5 μm) were deparaffinized by incubating slides at 60°C for 5 minutes followed by two changes of xylene substitute (Sigma #A5597). Sections were rehydrated with two changes of 100% and single changes of 95%, 70%, 50%, and 30% ethanol and deionized water, five minutes each. Chondroitinase ABC (4U/mL diluted 1:40 in Tris-HCl pH7.5) (Seikagaku #100332) treatment for 30 minutes at room temperature was used on sections for versican staining. Proteinase K (diluted 1:900 in Tris-HCl pH7.5) (Sigma #4850) treatment for 10 minutes at room temperature was used on sections prior to ADAMTS staining. Antigen Retrieval was performed by incubating slides in 10mM Na-Citrate, 1mM EDTA, pH6.0 solution at sub-boiling temperature for 30 minutes followed by 10 minutes cooling. Blocking was with 1% BSA/PBS or M.O.M. blocking reagent (Vector Laboratories BMK #2202) for 1 hour at room temperature. Primary antibodies were diluted in PBS or M.O.M. protein reagent and incubated at 4°C overnight. The following antibodies were used: versican against GAGβ domain (EMD Millipore #AB1033; rabbit polyclonal; 1:100); versican V0/V1 Neo (DPEAAE; Thermo Scientific #PA1–1748A; rabbit polyclonal; 1:100); fibronectin (a gift from Dr. Kenneth M. Yamada, NIDCR, NIH; rabbit polyclonal; 1:100); collagen I (Abcam #ab34710; rabbit polyclonal; 1:100); cartilage link protein 1 (Developmental Studies Hybridoma Bank #9/30/8-A-4; mouse monoclonal; 1:100); MF-20 (Developmental Studies Hybridoma Bank #MF20; mouse monoclonal; 1:100); ADAMTS1 (R&D Systems #AF5867; sheep polyclonal; 1:100); ADAMTS5 (Thermo Scientific #PA5–27165; rabbit polyclonal; 1:100); ADAMTS20 (abcam #ab60148; rabbit polyclonal; 1:100); Ki-67 (Cell Signaling #9449; mouse monoclonal; 1:100); and phospho-histone H3 Ser10 (Cell Signaling #3377; rabbit monoclonal; 1:100). After washing, sections were incubated for 2 hours at room temperature with FITC, Cy3- and Cy5-conjugated secondary Fab fragment antibodies (Jackson Laboratories; 1:200), or Rhodamine-conjugated peanut agglutinin (PNA) (Vector Lab #RL-1072; 1:100), Alexa Fluor 488-conjugated helix pomatia agglutinin (HPA) (Molecular Probes #L11271; 1:200), Alexa Fluor 488-conjugated wheat germ agglutinin (WGA) (Molecular Probes #W11261; 1:200). Nuclear counterstaining was done using Hoechst 33342 (Invitrogen #H3570; 1:10,000) for 5 minutes at room temperature. Stained sections were mounted and visualized on a Zeiss LSM 710 confocal microscope. Representative stacked section confocal images are shown in each figure. Images were analyzed by NIH ImageJ software and assembled in Photoshop.

### Western Blotting

Outflow tract tissue (OFT) isolated from littermate samples of *Galnt1* wild type and null animals were lysed in RIPA buffer with sonication. Samples used for western blots were treated with neuraminidase (10 mU) (Sigma #N-2876) at 37°C for 2 hours. Samples were analyzed by SDS-PAGE under denaturing conditions and transferred to nitrocellulose membranes. For Smad, MAPK and EGFR western blot analyses, membranes were blocked with 5% BSA-TBST and probed with phospho-Smad1/5 and phospho-MAPK (Erk1/2), and total-Smad1 and MAPK antibodies (Cell Signaling #9516P, #4376, #6944, #9102, respectively; rabbit monoclonal for first 3 antibodies and rabbit polyclonal for the fourth antibody; 1:1000), or EGFR antibody (Cell Signaling #4267; rabbit polyclonal antibody; 1:1000) and α-Tubulin antibody (Cell Signaling #2125; rabbit monoclonal antibody; 1:1000). After washing, membranes were probed with HRP conjugated anti-rabbit IgG (Cell Signaling; 1:2000). For western blots probed with lectins, membranes were blocked with Carbo-Free 1X Blocking (Vector #SP-5040) and probed with peroxidase-labeled PNA (Sigma #L7759; 1:1000). The HRP signal was detected using a chemiluminescent substrate (Thermo Scientific #34080) and analyzed with a Fuji imager. NIH ImageJ was used to measure band intensity and calculate ratios for pSmad1/5 to total Smad1, pMAPK to total MAPK, and EGFR to α-Tubulin (setting the ratio for wild type littermate controls to 1). For western blots used to assess the specificity of antibodies to ADAMTS1, ADAMTS5, and ADAMTS20, after electrophoretically separated OFT or heart lysates were transferred to nitrocellulose membranes were blocked with Sigma blocking buffer (Sigma #6429), probed with the primary antibodies as described in the previous section “Immunofluorescence”, and then processed as described above to detect HRP signal.

### Laser Capture Microdissection (LCM)

Wild type and null embryos were embedded into tissue freezing medium (Sakura Finetek USA #4583) and cryosectioned at 10 μm thickness. Sections were quick stained with 70% ethanol, 20 seconds; RNA free molecular water 5 dips; Hematoxylin (Sigma #MHS16) 30 seconds; RNA free molecular water 5 dips; 70% ethanol, 20 seconds; 90% ethanol, 20 seconds; 100% ethanol, 20 seconds; and xylene substitute until ready to use. LCM of outflow tract cushion tissue was performed using the ArcturusXT (Life Technologies).

### Gene Expression Analysis

RNA purification and quantification was performed according to manufacturer’s instructions (Norgen Biotek #17200). Using the RNA from LCM, cDNA was synthesized using iScript cDNA Synthesis Supermix (BioRad #170–8840). Primers for *Galnt*, *Adamts* and *Bmp/Smad* genes used were previously published and are available on request [8]. qPCR was performed for 40 cycles (95°C for 10 seconds and 62°C for 30 seconds) using SYBR-green PCR Master Mix, 1 ng of each cDNA sample and the CFX96 Real-Time system (Bio-Rad). Gene expression was normalized to *29S* ribosomal RNA. Reactions were run in triplicate, and each experiment was repeated three times.

### Quantification of Valve and Cushion Anomalies and Immunofluorescence

To quantify adult valve thickness, the widest portion of the cusps and leaflets of valves were measured using a eSlide capture device, (Aperio ScanScope CS2), in collaboration with Aperio ImageScope software and NIH ImageJ software. Four independent measurements were taken per cusp or leaflet. The values were averaged. A minimum of four animals were used per genotype for statistical analysis.

To quantify embryonic OFT cushion (E10.5—E12.5) and valve thicknesses (E14.5), the images from widest portion of cushion tissues with a minimum depth of 20 μm were used. NIH ImageJ software was applied for measurement of OFT cushion cell number and area size using images captured from either Aperio ScanScope CS2 (H&E staining) or Zeiss LSM 710 confocal microscope (nuclei counterstaining). The values were averaged and a minimum of three littermate animals were used per genotype from three crosses for statistical analysis.

Fluorescent intensity of confocal images was measured using NIH ImageJ software. To quantify versican (total and DPEAAE), staining at the OFT cushion tissue (E12.5), or valves (E14.5) was measured and then compared to total nuclei staining in the area of interest. To quantify cartilage link protein, fibronectin, and collagen I, the fluorescent intensity in the OFT tissue (E11.5, E12.5) was compared to that of the myocardium. The ratio of *Galnt1^-/-^* was compared and normalized to a wild-type ratio of 1. A minimum of three independent crosses to generate wild type and *Galnt1* null littermates were performed in the analysis. To quantify the cell proliferation markers pH3 and Ki-67, the total number of positive nuclear pixels above a background threshold was counted and normalized to the total number of cushion mesenchymal nuclei; the ratio for wild type littermate controls was set to 1.

### Statistical Analyses

Values were plotted as means ± standard errors of the mean for each group from three or more experiments. Student’s *t*-test was used to calculate *P*-values (2 tailed, type 2).

## Results

### Adult *Galnt1^-/-^* Mice Display Left Ventricular Hypertrophy, Cardiac Chamber Dilation and Valve Thickening

*Galnt1* null mice were generated by crossing floxed *Galnt1* mice to Zp3-Cre transgenic mice, which results in excision of exon 3 of *Galnt1* gene in all tissues, as described previously [10]. The genotypes of E12.5 embryos from *Galnt1* heterozygous mating followed close to the expected Mendelian ratio (*+/+*, *+/-*, -*/-* = 1:2.4:0.91; total E12.5 embryos genotyped = 289). However, the pups genotyped at weaning had reduced proportions of homozygous null animals (*+/+*, *+/-*, -*/-* = 1:1.875:0.67; total number of pups genotyped = 624). Thus, about 26% of homozygous null animals appear to die in utero beyond E12.5 or before the age of one month, for reasons yet unknown. In rare instances, adult female *Galnt1* null mice were observed to die suddenly, typically within days of giving birth.

**Figure 1:**
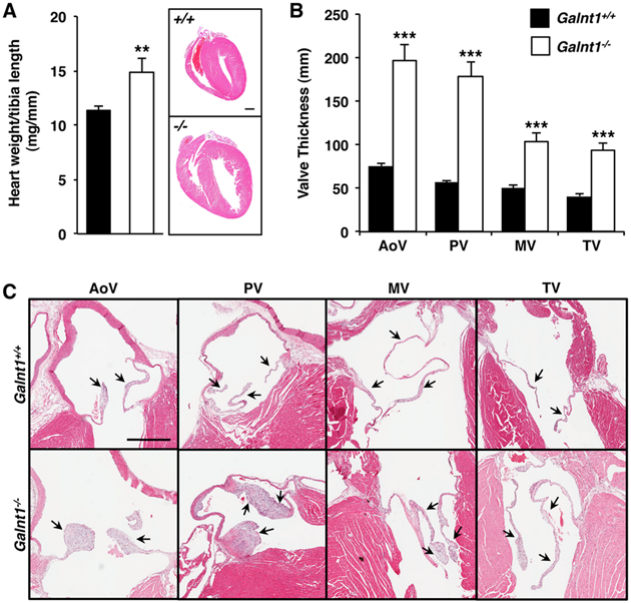
Cardiac dilation and valve thickening in adult *Galnt1* null mice. (A) Loss of *Galnt1* results in an increased heart size in 4-to-5-month-old *Galnt1* nulls (*-/-*) (n = 11) versus wild type (*+/+*) (n = 10) littermates by heart weight-to-tibia length ratios. A sagittal section of hematoxylin/eosin (H&E) staining of 4-to-5-month-old heart shows the dilation of heart chamber in *Galnt1* null. (B) Loss of *Galnt1* results in thickened valves relative to wild type controls. Mean valve thickness of 4-to-5-month-old littermates show significantly thickened aortic (*Galnt1^+/+^* n = 19; *Galnt1^-/-^* n = 19), pulmonary (*Galnt1^+/+^* n = 18; *Galnt1^-/-^* n = 19), mitral (*Galnt1^+/+^* n = 16; *Galnt1^-/-^* n = 16), and tricuspid (*Galnt1^+/+^* n = 17; *Galnt1^-/-^* n = 15) valves in *Galnt1^-/-^* relative to wild type (*+/+*). (C) H&E staining of 4- to 5-month-old hearts showing aortic, pulmonary, mitral, and tricuspid valves; arrows point to valve leaflets. Student’s t-test was used to calculate P-valves. **, P < 0.01; ***, P < 0.001. AoV, aortic valve; PV, pulmonary valve; MV mitral valve; TV, tricuspid valve. Scale bar A = 1mm, C = 500 μm.

Anatomic analysis of adults revealed enlarged hearts in the *Galnt1^-/-^* mice relative to wild type controls (Fig. 1A). No obvious abnormalities in other organs (with the exception of the submandibular glands [8]) were observed. The heart weight-to-tibia length ratio of 4-to-5-month-old *Galnt1* null mice was greater than those of wild type (Fig. 1A). In addition, semilunar (SL) valves (aortic and pulmonary valves) and atrioventricular (AV) valves (mitral and tricuspid valves) were thickened in adult *Galnt1* nulls relative to wild type controls (Fig. 1B, C). Due to the higher penetrance and severity of the SL valve thickening, most embryonic analysis was performed on these valves.

### Cardiac Function is Compromised in Adult *Galnt1^-/-^* Mice

**Figure 2:**
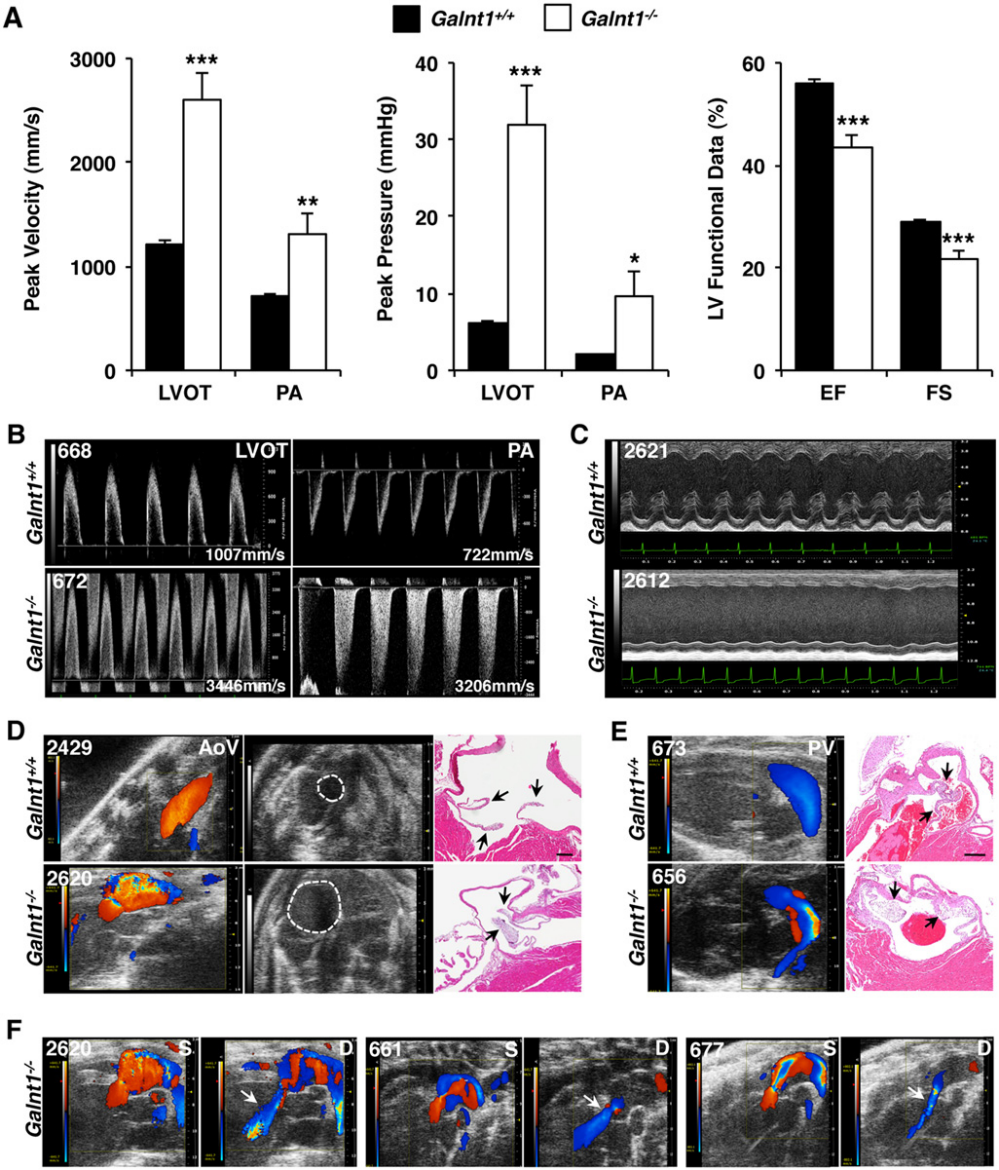
Cardiac function is compromised in *Galnt1* null mice. (A) Pulsed wave Doppler data from major cardiac vessels in 4-to-5-month-old adult mice. *Galnt1^-/-^* mice have significantly increased peak velocity across both the left ventricular outflow tract and pulmonary artery. *Galnt1^-/-^* mice have a significant increase in the peak pressure gradient across the left ventricular outflow tract and the pulmonary artery. In 2-D M-mode images of the left ventricle at mid-papillary level, the left ventricular ejection fraction and fractional shortening are both significantly reduced in *Galnt1^-/-^* mice. (B) Pulsed wave Doppler velocity tracings in the left ventricular outflow tract and pulmonary artery illustrate the increased velocity in null mice (A). (C) 2-D M-mode image through the LV. The contractility of the LV is impaired in the *Galnt1^-/-^* mice compared with wild type. (D) Color Doppler images of blood flow in the ascending aorta along with the animals’ transverse 2-D image at the cardiac base of wild type and null animals, and corresponding H&E staining of the valve; arrows point to valve leaflets. In the color Doppler images, the color pattern observed in the vessels of the *Galnt1^-/-^* mice indicates disturbed, turbulent and high velocity blood flow compared to the uniform coloration in the corresponding images from the wild type mice representing laminar flow. In the transverse 2-D image, dashed lines indicate perimeter of ascending aorta, and show severe post-stenotic dilation in the *Galnt^-/-^* mouse as compared to the normal, non-dilated aorta in the *Galnt^+/+^* mouse. (E) Similar disrupted flow in *Galnt1^-/-^* mice can be seen in color Doppler images of blood flow in the pulmonary artery, which have been matched to H&E staining of the valve. (F) Color Doppler images of aortic flow in *Galnt1^-/-^* showing disrupted systolic (S) flow followed by diastolic (D) aortic regurgitation (white arrow). Animal numbers, upper left, in echocardiography images are matched to S1 Table measurement data. Student’s t-test was used to calculate P-valves. *, P < 0.05; **, P < 0.01; ***, P < 0.001. LVOT, left ventricular outflow tract; PA, pulmonary artery; AoV, aortic valve; PV, pulmonary valve. Scale bars: D, E = 200 μm.

We next investigated the functional consequences of the valvular thickening in the hearts of *Galnt1^-/-^* mice. Using echocardiograph imaging, we simultaneously assessed the aortic and pulmonary arteries by pulsed-wave (PW) and color Doppler; and cardiac function by M-mode in mice whose valves were also subsequently analyzed by H&E staining (Fig. 2A–F; see S1 Table). Compared to wild type (*Galnt1^+/+^*, n = 17; *Galnt1^-/-^*, n = 19), seventeen out of nineteen null mice (89.5%) had increased transvalvic velocity and pressure gradients across either the aortic and/or the pulmonic valves. Thirteen out of nineteen (68.4%) null mice had increased transvalvic velocity and pressure gradients across the aortic valve, and eight out of nineteen (42.1%) null mice had increased transvalvic velocity and pressure gradients across the pulmonary valve. Furthermore, two-dimensional M-mode echocardiography of these mice also revealed depressed left ventricular ejection fraction (LVEF) and fractional shortening (FS); increased left ventricle (LV) end systolic and diastolic volumes; increased diastolic and systolic LV chamber dimensions; and increased LV anterior and posterior diastolic and systolic wall dimensions in the null mice compared to the wild type littermate controls (Fig. 2A–C; see S2 Table). Additionally, color Doppler imaging showed turbulent blood flow through either the aortic and/or pulmonary valves in eighteen out of nineteen *Galnt1* null mice (Fig. 2D–E), and seven cases of regurgitation (36.8%) through aortic valves into the LV were also observed in *Galnt1* null mice (Fig. 2F). Finally, the transverse 2D image through the aorta (dotted line) at the cardiac base shows severe post-stenotic dilation as measured by an aortic cross section diameter of 2.8 mm in the *Galnt1* null mouse compared to 1.4 mm in the wild type littermate control mouse (Fig. 2D). These results suggest the occurrence of stenosis in both aortic and pulmonary valves leads to the development of cardiac chamber dilation and reduced ventricular function (summarized in S2 Table). Taken together, echocardiography results demonstrate that all *Galnt1* null mice that had evidence of valvular stenosis also had cardiac functional impairments. Results for various measures of functional impairment and histological measurements across individual animals are reported in S1 Table.

### *Galnt1* Deficiency Affects Valve Development

**Figure 3:**
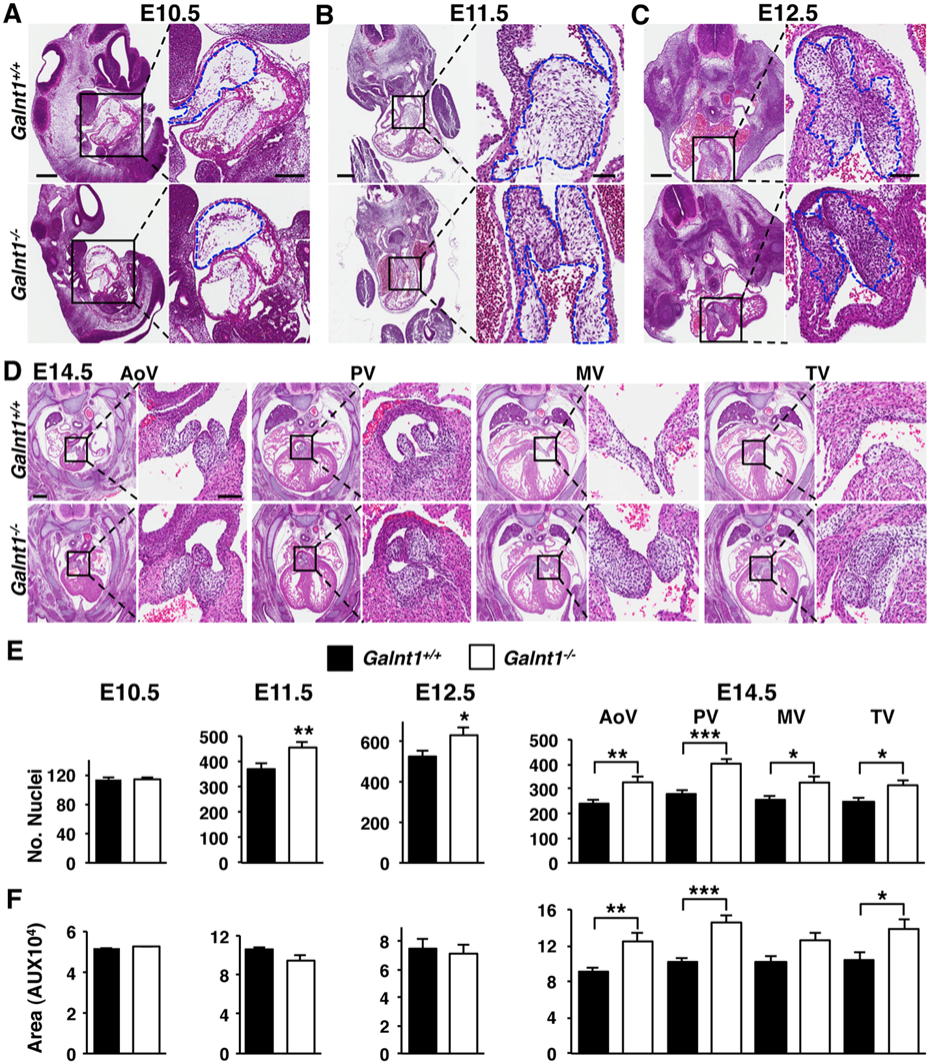
Increased heart cushion cell number is seen in valve cusps of *Galnt1^-/-^* mice. H&E staining of wild type and *Galnt1* null littermate outflow tract (OFT) cushions and atrioventricular cushions at E10.5 (A), OFT cushions at E11.5 (B), and OFT cushions at E12.5 (C). (D) HE staining of AoV, PV, MV and TV at E14.5. Images in B–D are of transverse histological sections and images in A are of sagittal sections. The boxed region is magnified to the right of each image. Blue dash lines in higher magnification of A–C highlight developing cushion area. (E) Mean number of nuclei in the developing OFT cushions, measured over 20–40 μm sections (E10.5-E12.5) and heart valves (E14.5) shows a significant increase in cell number in *Galnt1^-/-^* starting at E11.5 (*Galnt1^+/+^* and *Galnt1^-/-^*, n = 3), persisting through E12.5 (*Galnt1^+/+^* and *Galnt1^-/-^*, n = 5), and E14.5 (*Galnt1^+/+^* and *Galnt1^-/-^* AoV and PV, n = 11; *Galnt1^+/+^* and *Galnt1^-/-^* MV and TV, n = 8). No significant change is seen in cell number at E10.5 OFT (*Galnt1^+/+^* and *Galnt1^-/-^*, n = 3). (F) Size quantification (by NIH ImageJ) of developing OFT cushions at E10.5 (*Galnt1^+/+^* and *Galnt1^-/-^*, n = 4), E11.5 (*Galnt1^+/+^* and *Galnt1^-/-^*, n = 5), E12.5 (*Galnt1^+/+^* and *Galnt1^-/-^*, n = 12), and all four valves at E14.5 (*Galnt1^+/+^* and *Galnt1^-/-^* AoV and PV, n = 11; *Galnt1^+/+^* and *Galnt1^-/-^* MV and TV, n = 8). No differences in size of OFT cushion tissue is seen at E10.5, E11.5, or E12.5; however, at E14.5 a significant increase in valve size can be seen in the AoV, PV, and TV. Student’s t-test was used to calculate P-valves. *, P < 0.05; **, P < 0.01; ***, P < 0.001. AoV, aortic valve, PV, pulmonary valve, MV mitral valve, TV, tricuspid valve. Scale bars: A, B, C, D = 300 μm for low magnification and 100 μm for higher magnification view.

To examine if the valve thickening observed in adults is the result of a developmental defect, we next examined embryonic valve development in *Galnt1^-/-^* embryos. SL valve development begins at E9.5 as neural crest-derived cells migrate into the cardiac jelly and endocardial cells undergo epithelial to mesenchymal transition (EMT) to form the mesechymal cushion known as the outflow tract (OFT) cushion [5, 7]. After EMT concludes at E10.5, the valve progenitor cells of the endocardial cushion undergo cell proliferation and increased synthesis of extracellular matrix (ECM) components at E11.5 and E12.5 [11]. Histological analysis of E10.5 revealed no obvious OFT cushion abnormalities (Fig. 3A), and no significant difference between *Galnt1^-/-^* and wild type embryos in cushion cell number or cushion area (Fig. 3E, F), indicating that EMT is occurring normally in *Galnt1^-/-^* embryos. At E11.5 and E12.5, the cushion cell numbers were significantly increased, while cushion area remained unchanged in *Galnt1^-/-^* compared to wild type littermate controls (Fig. 3B, C, E, F). At E14.5, when valves become morphologically distinct from the surrounding tissues [11], there was a significant increase in cell numbers within all 4 valves in the *Galnt1^-/-^* embryos (Fig. 3D, E). Additionally, the aortic valve, pulmonary valve, and tricuspid valve (AoV, PV, TV, respectively) show a statistically significant increase in overall valve size at E14.5 in *Galnt1^-/-^* embryos (Fig. 3F).

**Figure 4:**
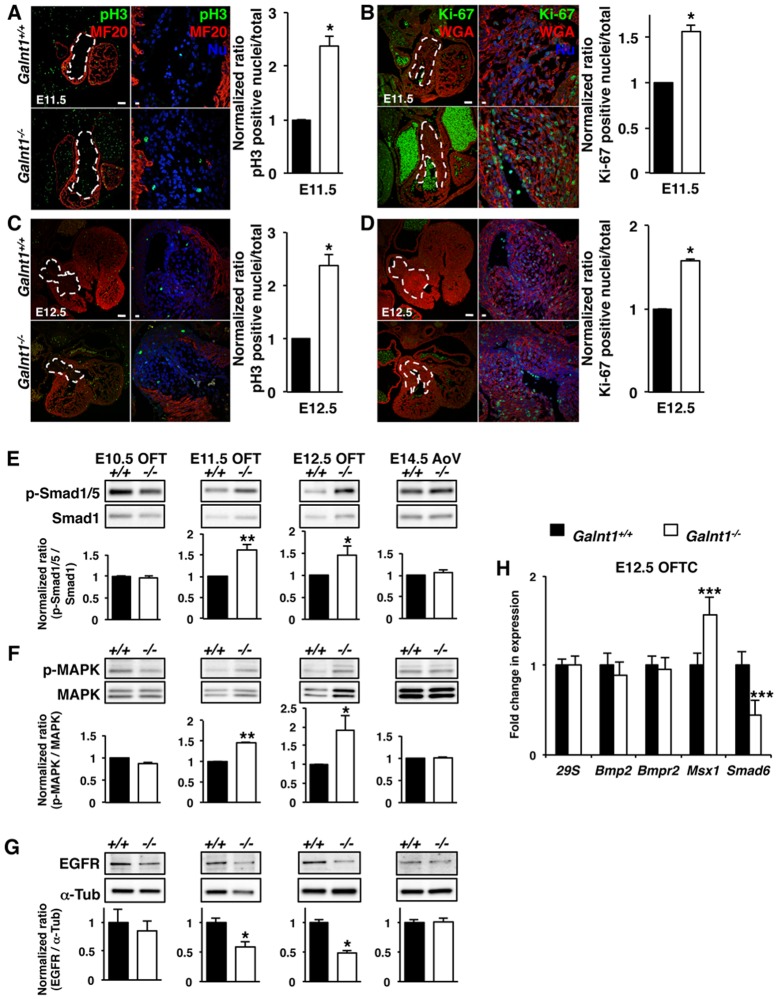
Loss of *Galnt1* causes increased cell proliferation during early valve development. (A–D) Confocal images and quantification of phospho-histone H3 (pH3) and Ki-67 proliferation markers in developing valves at E11.5 and E12.5. (A) E11.5 OFT cushion tissue (pH3, green; myocardial marker MF20, red; n = 4). (B) E11.5 OFT cushion tissue (Ki-67, green; cell surface marker lectin WGA, red; n = 3). (C) E12.5 OFT cushion tissue (pH3, green; myocardial marker MF20, red; n = 3). (D) E12.5 OFT cushion tissue (Ki-67, green; cell surface marker lectin WGA, red; n = 3). White dash lines in lower magnification in A–D highlight developing cushion area. Higher magnification views are shown to the right of each image. Percentage of nuclei (Nu; blue) positive for pH3 or Ki-67 in wild type samples was set to 1 and relative differences in *Galnt1* nulls is graphed to the right of each image. Western blotting reveals an increase in phosphorylation of (E) Smad1/5 (BMP) and (F) MAPK (ERK1/2), (G) decreased EGFR in *Galnt1* null OFT samples relative to wild type at E11.5 (n = 4), and E12.5 (n = 6) while remaining unchanged in E10.5 OFT (n = 3) and in E14.5 AoV (n = 3). Samples were normalized to total Smad1, total MAPK and α-Tubulin (α-Tub). (H) Expression of Bmp/Smad genes was examined by qPCR in E12.5 OFT cushion (OFTC) tissues (triplicates, n = 5) isolated by laser-capture microdissection (LCM). While expression of *Bmp2* and *Bmpr2* remains unchanged, gene expression of *Msx1* (a *Bmp2* downstream target gene) is significant increased, and *Smad6* expression (an inhibitor of BMP signaling) is greatly reduced. Student’s t-test was used to calculate P-valves. *, P < 0.05; **, P < 0.01; ***, P < 0.001. Scale bars: A, B, C, D = 100 μm for low magnification and 10 μm for higher magnification view.

To identify when increased cell proliferation begins in the *Galnt1^-/-^* valves, we stained valves for markers of active mitosis (phospho-histone H3 (pH3) and Ki-67). Interestingly, we found a statistically significant increase in both pH3-postitive and Ki-67-positive cushion cells in the OFT of *Galnt1^-/-^* embryos at both E11.5 and E12.5 (Fig. 4A–D). These findings suggest that the increased cell proliferation in *Galnt1^-/-^* valves is occurring at E11.5, resulting in increased cell numbers and increased valve size by E14.5.

Cell proliferation during heart valve morphogenesis is regulated by a number of signaling pathways, including transforming growth factor beta (TGF-beta) associated bone morphogenetic protein (BMP) signaling, mitogen-activated protein kinase (MAPK) signaling and epidermal growth factor receptor (EGFR) signaling [2, 3, 12]. In *Galnt1^-/-^* OFTs, we detected a specific increase in BMP signaling (via phosphorylated Smad1/5) and MAPK signaling (via phosphorylated ERK1/2) at both E11.5 and E12.5 (Fig. 4E, F). No phosphorylation differences were seen between wild type and *Galnt1^-/-^* OFTs at E10.5 or at E14.5 (Fig. 4E, F). Lending further support to increased BMP signaling, we also detected increased expression of the BMP2 downstream target gene, *Msx1* [3] (Fig. 4H). However, no alterations in gene expression of either *Bmp2* or *Bmpr2* were detected (Fig. 4H). EGFR signaling can act as an antagonist to BMP signaling by activating the expression of the BMP-inhibitory factor, *Smad6* [7, 13, 14]. Consistent with the increased BMP signaling seen in the *Galnt1^-/-^* OFTs, we found a decrease in EGFR protein levels in both E11.5 and E12.5 *Galnt1^-/-^* OFTs, along with a corresponding decrease in gene expression of *Smad6* (Fig. 4G, H). These results demonstrate that loss of *Galnt1* causes a specific increase in the conserved BMP and MAPK signaling pathways at E11.5-E12.5 that result in increased cell proliferation and larger valves at E14.5.

### *Galnt1* Affects Multiple Proteins in the Developing Valve

**Figure 5:**
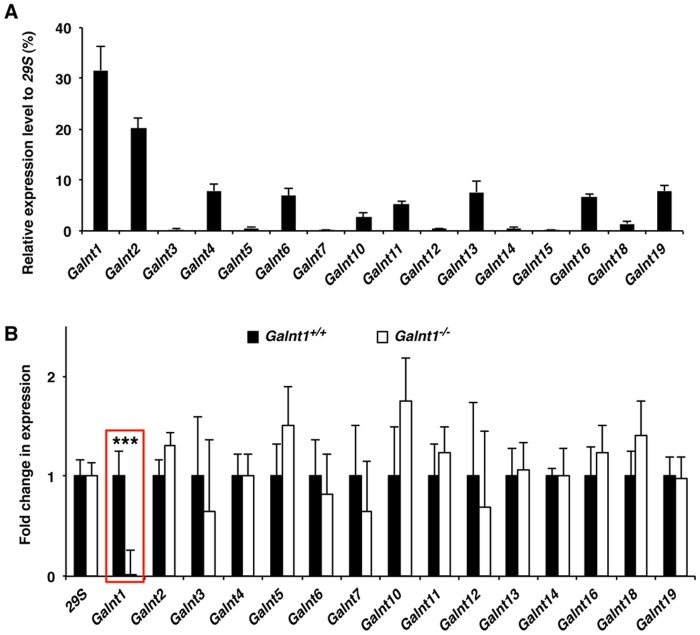
*Galnt1* is the most highly expressed isoform in the forming cardiac valve. (A) *Galnt* transcript levels in E12.5 OFT cushion samples (triplicates, n = 3) isolated by LCM. (B) Loss of *Galnt1* expression (highlighted by red box) in LCM OFT cushion from *Galnt1*-deficient mice was verified by qPCR. Expression of other *Galnt* family members found to be unchanged in *Galnt1* null (triplicates, n = 3). Expression was normalized to *29S*. ***, P < 0.001.

**Figure 6:**
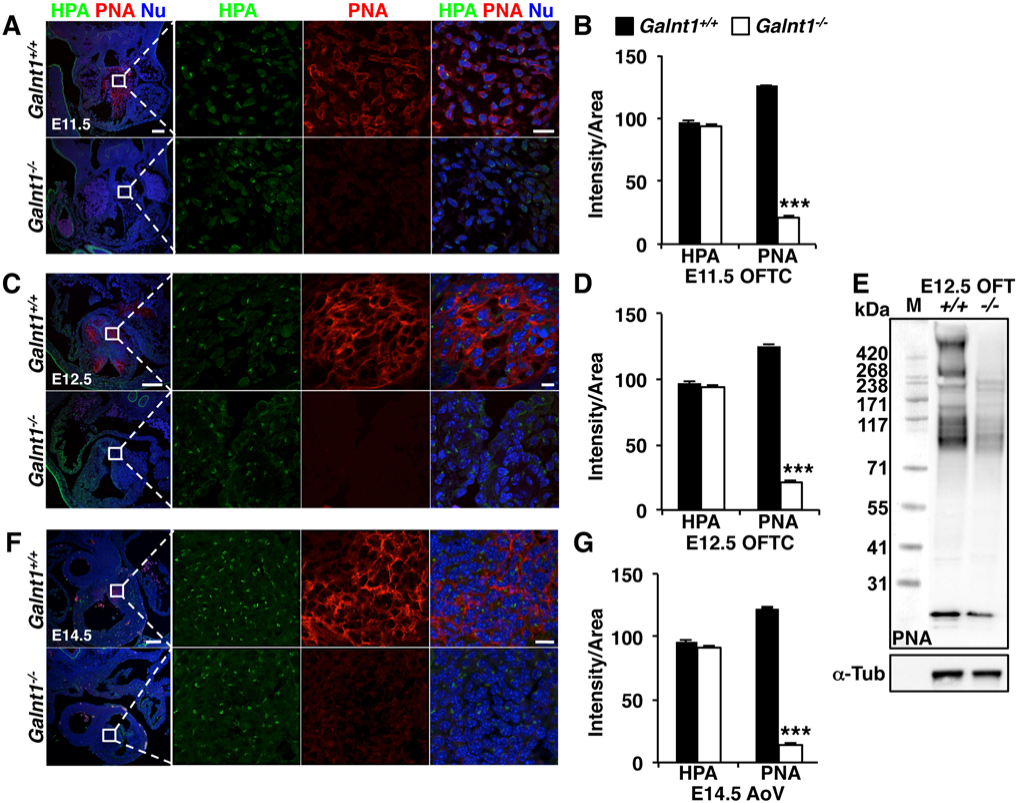
O-glycan expression is diminished in *Galnt1^-/-^* developing valve tissue. Confocal images of HPA (green) labeling Tn antigen, PNA (red) labeling T antigen, and nuclei (Nu; blue) display a selective reduction in PNA staining in (A) E11.5 OFT cushion, (C) E12.5 OFT cushion, and (F) E14.5 developing valves (AoV pictured). Boxed region is magnified to the right of each image. Quantification of HPA and PNA fluorescent signal intensity by ImageJ software for (B) E11.5 OFT cushion (HPA, n = 2; PNA, n = 3), (D) E12.5 cushion (HPA, n = 3; PNA, n = 6), and (G) E14.5 AoV (HPA, n = 2; PNA, n = 3) is shown. (E) Western blot of E12.5 OFT cushion proteins probed with PNA. Samples were normalized to α-Tubulin (α-Tub). Student’s t-test was used to calculate P-valves. ***, P < 0.001. AoV, aortic valve. OFTC, outflow tract cushion. Scale bars: A, C, F = 100 μm for low magnification and 10 μm for higher magnification view.

We next set out to identify the substrates of *Galnt1* that are responsible for the changes in conserved signaling pathways and cell proliferation observed during valve development. During embryonic stages, *Galnt1* is the most abundant member of the *Galnt* family expressed in the developing valve tissue, and loss of *Galnt1* does not affect expression of other *Galnts* (Fig. 5A, B). Wild type valves are abundantly stained with lectins (PNA) that detect O-linked glycans (Fig. 6). However, all PNA staining normally present during valve development (from E10.5-E14.5) is lost in *Galnt1* nulls, whereas HPA staining does not change (Fig. 6A–D, F–G), indicating that *Galnt1* is responsible for forming the valve-specific O-glycoproteins at these stages. Interestingly, western blots comparing O-glycosylated proteins from E12.5 OFTs from wild type and *Galnt1^-/-^* revealed not one, but many O-glycosylated proteins altered upon loss of *Galnt1* (Fig. 6E). These results suggest that the phenotypes seen in the *Galnt1* nulls likely represent the aggregate effects of aberrant glycosylation of multiple proteins expressed in the developing heart valves.

**Figure 7:**
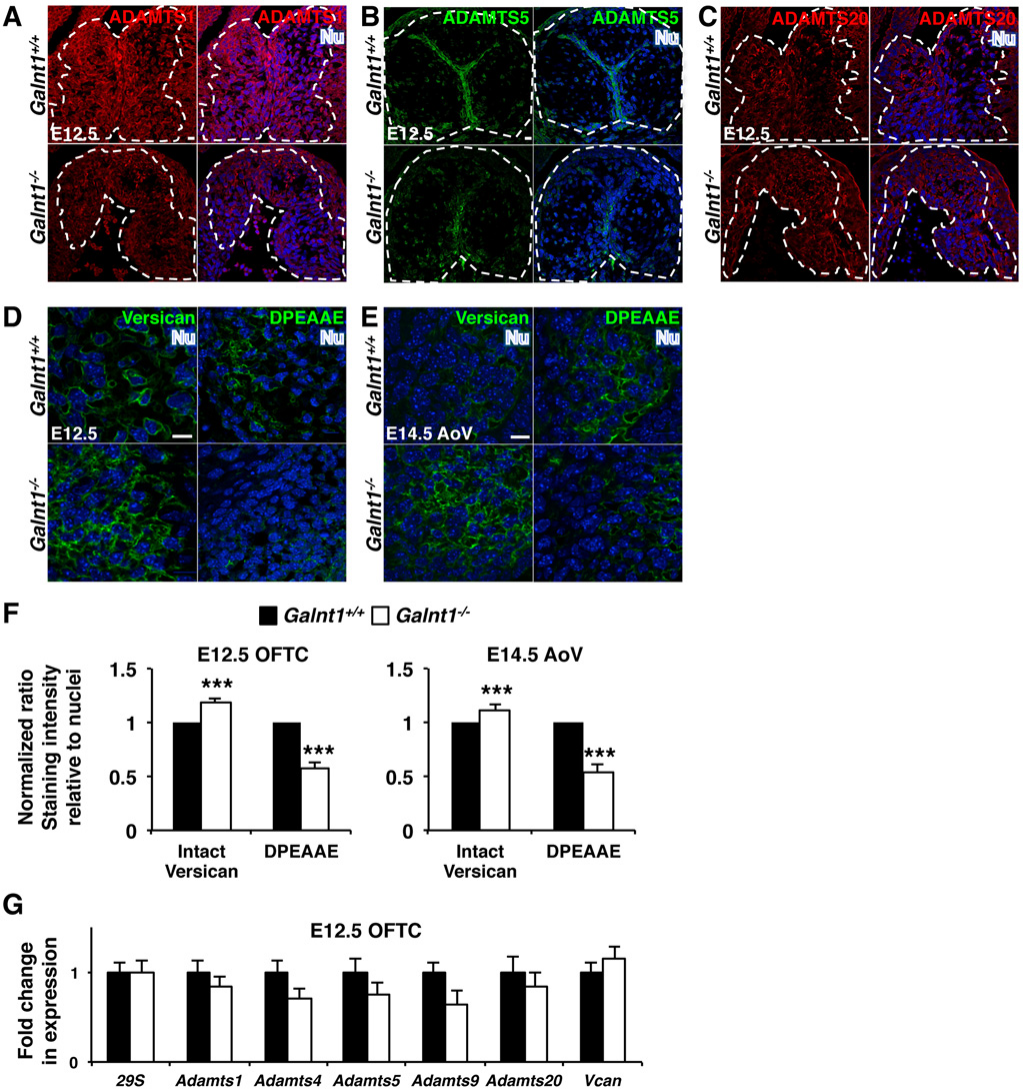
Diminished ADAMTS and reduced versican cleavage in *Galnt1* nulls. (A) ADAMTS1, expressed in the myocardium and developing cardiac cushion tissue (white dashed lines), is decreased at E12.5 in *Galnt1^-/-^* relative to wild type (*Galnt1^+/+^*). (B) ADAMTS5, expressed in the myocardium and hinge region of developing valve leaflets (white dashed lines), is diminished at E12.5 and E14.5 in *Galnt1^-/-^* relative to wild type (*Galnt1^+/+^*). (C) ADAMTS20, expressed in the myocardium and developing cardiac cushion tissues (white dashed lines), is unchanged at E12.5 in *Galnt1^-/-^*. (D) Confocal images of intact versican (versican, green), (E) cleaved versican (DPEAAE; green), and nuclei (Nu; blue) show an increase in intact versican accompanied by a decrease in cleaved versican in E12.5 OFT cushions and E14.5 AoV of *Galnt1^-/-^* relative to wild type. (F) Fluorescent staining intensity of intact versican at E12.5 (n = 5) and E14.5 (n = 5) and cleaved (DPEAAE) versican at E12.5 (n = 4) and E14.5 (n = 4) relative to nuclei staining, and normalized to wild type (equal to 1), at E12.5 and E14.5 shows significant increase in *Galnt1^-/-^* OFT cushion tissue. (G) qPCR analysis of expression of major *Adamts* genes and *versican* (*Vcan*) from E12.5 OFT cushion (isolated by LCM) is unchanged in *Galnt1* nulls relative to wild type (triplicates, n = 4). ***, P < 0.001. AoV, aortic valve. OFTC, outflow tract cushion. Scale bars: A, B, C, D, E = 10 μm.

To further investigate what specific proteins may be affected by the loss of *Galnt1*, we examined proteins that are known to be important for valvulogenesis. The ADAMTS proteases are required for proper valve development [15, 16, 17]. We therefore evaluated expression of members of the ADAMTS family in developing valves of wild type and *Galnt1* null animals by qPCR and immunofluorescence (The specificity of antibodies used to detect ADAMTS1, ADAMTS5, and ADAMTS20 were validated by western blot analysis and showed apparent molecular masses that are consistent with previous reports [18–22] (see S1 Fig.). While we found no changes in *Adamts1*, *4*, *5*, *9* and *20* gene expression by qPCR in E12.5 *Galnt1^-/-^* OFT cushion (Fig. 7G), we did observe a reduction in ADAMTS1 and ADAMTS5 protein in developing valves of *Galnt1* nulls (Fig. 7A, B). No changes in protein abundance were seen for ADAMTS20 (Fig. 7C). These results suggest that reduced levels of specific proteases may be contributing to the phenotypes seen in the absence of *Galnt1*.

As ADAMTS1 and ADAMTS5 are required for processing the proteoglycan versican during cardiac development, we next examined versican processing using antibodies to stain for total versican (intact and cleaved) and cleaved versican (DPEAAE neoepitope) only [15, 23]. As seen in Fig. 7D, E and F, a dramatic reduction in versican processing (i.e. a decrease in cleaved versican with an increase in total versican) was observed *Galnt1^-/-^* embryos. These results suggest that the reduction of ADAMTS1 and ADAMTS5 in *Galnt1* nulls results in alterations in versican processing during cardiac development.

**Figure 8:**
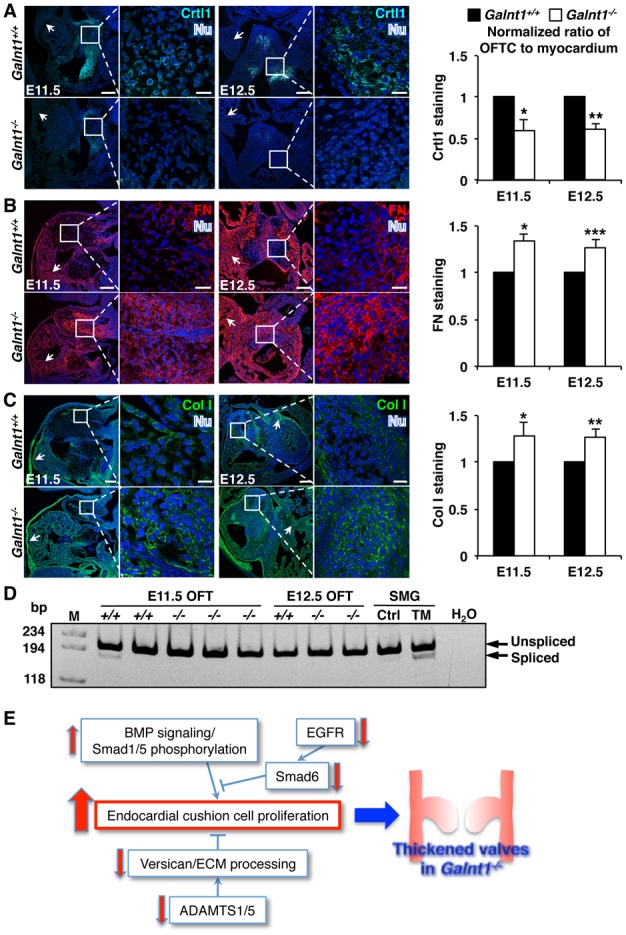
Extracellular matrix (ECM) protein accumulation is altered in *Galnt1* nulls. Confocal images of (A) cartilage link protein 1 (Crtl) at E11.5, and E12.5; (B) fibronectin (FN) at E11.5, and E12.5; (C) collagen I (Col I) at E11.5, and E12.5 are shown in OFT cushions. A magnified view of the cushion is shown in the boxed inset. Significant changes in fluorescent staining intensity in outflow tract cushion tissue compared to myocardial staining intensity, is seen at E11.5 (n = 3) and E12.5 (n = 4) null mice. Ratio of OFT to myocardial staining was calculated and then *Galnt1^-/-^* was normalized to wild type = 1. In A–C, arrows indicate areas of myocardium that show similar staining intensity levels from wild type to null. Nu, nuclei (blue). OFTC, outflow tract cushion. Scale bars: A, B, C, = 100 μm for low magnification and 10 μm for higher magnification views. (D) Developing cardiac heart tissue does not show indications of ER stress by Xbp1 mRNA splicing. RNA was extracted from *Galnt1^+/+^* and *Galnt1^-/-^* OFT at E11.5 (*Galnt1^+/+^* and *Galnt1^-/-^*, n = 3) and E12.5 (*Galnt1^+/+^* n = 3; *Galnt1^-/-^* n = 6), and reverse transcribed to synthesize cDNA. 10 ng cDNA was used for qPCR. 10 ng/ml tunicamycin (TM) treated E12.5 mouse submandibular gland (SMG) was used as positive control, and DMSO treated glands as negative control (Ctrl). Water (H_2_O) was used for no cDNA negative control. OFT, outflow tract. (E) Model of pathways and changes seen in the *Galnt1^-/-^* OFTs that may lead to increased endocardial cell proliferation and valve thickening. Red arrows indicate the changes observed in *Galnt1* nulls relative to wild type.

We also examined the expression of other ECM proteins known to be involved in cardiac development. Cartilage link protein 1 (Crtl1) [24] was dramatically reduced in the OFT cushion of *Galnt1* nulls relative to wild type littermates at E11.5 and E12.5 (Fig. 8A). Additionally, we found increased accumulation of other ECM proteins in the developing valves of *Galnt1* nulls relative to wild type littermates, including collagen I and fibronectin (Fig. 8B, C). Previous work in the developing salivary glands in *Galnt1^-/-^* mice demonstrated decreased secretion of ECM components and an induction of ER stress. However, we did not detect any increase in markers of ER stress in the developing heart tissue (Fig. 8D). Therefore, loss of *Galnt1* in the developing valves results in the aberrant glycosylation of many proteins, as well as changes in the cleavage and abundance of many factors that are known to regulate valvulogenesis, ultimately leading to valvular stenosis and cardiac impairment.

## Discussion

Our study demonstrates for the first time that *Galnt1* expression is required for normal heart valve development. This requirement is specific for *Galnt1* since other *Galnts* are expressed in the OFT and their presence does not compensate for the loss of *Galnt1*. In this study, we found evidence for altered proteases levels, altered ECM processing/abundance and changes in BMP and MAPK signaling, all of which could contribute to the increase in endocardial cushion cell proliferation observed in *Galnt1* nulls (Fig. 8E). Given the complexity of cardiac valve formation and the number of normally O-glycosylated proteins that change upon loss of *Galnt1*, it is not surprising that our study points to multiple effects of ablating *Galnt1* that together, could contribute to the resultant phenotypes observed (Fig. 8E).

The development of the SL valves begins at E9.5 with the migration of neural crest-derived cells into the cardiac jelly and EMT, which concludes at E10.5. Subsequent stages are dependent upon the synthesis and processing of ECM proteins that regulate cell proliferation and the eventual remodeling of the OFT to form the mature valves [25, 26]. Interestingly, O-glycans are normally abundant in the OFT at E11.5, at which time ECM synthesis and cell proliferation are beginning to occur. However, in *Galnt1* nulls, we observed loss of O-glycans as well as changes in the proliferative capacity of cells within the OFT cushion. During early heart morphogenesis, TGF-beta associated BMP signaling is critical for normal cushion cell proliferation through Smad phosphorylation [3]. In *Galnt1* null animals, we detected a specific increase in BMP and MAPK signaling at E11.5 and E12.5, as well as changes in factors that regulate the BMP pathway. Recent studies have revealed that certain signals are required to restrict BMP and MAPK signaling during valve development to limit cell proliferation [7, 12, 27, 28]. For example, mutations that increase BMP/Smad signaling resulted in increased cell proliferation and thickened valves, similar to what is seen in this study [2, 13]. Taken together, our data suggests a loss of *Galnt1* disrupts the balance of conserved signaling pathways, thereby affecting cell proliferation during valve development.

In addition to changes in BMP and MAPK signaling, we detected changes in the level of proteases known to remodel the matrix as well as changes in the abundance of certain matrix proteins. Decreases in ADAMTS1 and ADAMTS5, both of which are known to cleave versican [15, 16, 23], are likely responsible for decreased versican cleavage seen in the developing valves of *Galnt1* nulls. As versican remodeling is required for proper cardiac development [15, 16], these changes likely contribute to the valve phenotypes observed. Indeed, mice deficient for ADAMTS5 phenocopy the valve defects observed in this study [16]. Additionally, the phenotype of *Adamts*5 nulls was partially rescued by reducing levels of intact versican [16], further supporting the notion that versican processing is crucial for proper heart development. How the processing/remodeling of other ECM proteins affects valve development is an area of ongoing investigation. Many proteoglycans/ECM proteins are thought to maintain local growth factor concentration, as well as provide structural cues. Thus, changes in the matrix could lead to alterations in the structure of the microenvironment and/or signaling cascades that could contribute to increased cell proliferation seen in *Galnt1* nulls. Inappropriate “over expression” of various matrix proteins could lead to aberrant growth factor-mediated signaling [6, 16, 26, 29, 30] that could explain, in part, the enhanced activation of cell signal cascades that may be responsible for the increases in cell number observed in the developing cushions.

Previous work from one of our labs demonstrated decreased ECM protein secretion and an induction of the endoplasmic reticulum stress response in developing submandibular glands (SMG) of *Galnt1* null animals, with a concomitant decrease in MAPK signaling and cell proliferation [8]. In the developing cardiac valves of these animals, we find quite the opposite, as signaling pathways and cell proliferation are increased and no evidence of ER stress is detected. The differences observed between these two tissues in the *Galnt1* nulls may be reflective of the unique developmental processes specific to epithelial and mesenchymal tissues, as well as unique roles played by O-glycosylation in the synthesis, secretion and/or remodeling of ECM proteins in these diverse cells types.

The aberrant development of cardiac valves in *Galnt1* mice leads to compromised cardiac function in adult animals. Over time, chronic exposure to aortic and/or pulmonic stenosis led to expected sequelae including post stenotic dilation of affected vessels, regurgitation, cardiac chamber dilation, and impaired ventricular ejection fractions and fractional shortening. Therefore, the *Galnt1* null animals may provide insight into congenital heart dysfunction as well as represent a new model to study cardiac valve repair or regeneration [31, 32].

## Supporting Information

S1 Table Histological and echocardiography data from each animal analyzed at 4–5 months allows for individualized direct comparison of morphological and functional changes. Wild type (n = 17) and *Galnt1-/-* (n = 19) valve thickness measurements (AoV, PV, MV, TV) from histological sections and comparison of PA and LVOT pulsed waved Doppler measurements and LV functional data in adult mice. Bold font indicates impairment seen by echocardiography, based on normal measurements for wild type (B6) mice imaged at the NHLBI mouse facility (bottom). AoV, aortic valve, PV, pulmonary valve, MV mitral valve, TV, tricuspid valve; LV, left ventricle; RV, right ventricle; LVOT, left ventricle outflow tract; PA, pulmonary artery; EF, ejection fraction; FS, fractional shortening.
(XLSX)

S2 Table Comparison of LV function and arterial pressure gradients in mice with aortic and/or pulmonic stenosis to mice with no valvular lesions. Mice with valvular stenosis demonstrate severely elevated peak pressure gradients near the affected valves and a corresponding decline in left ventricular function. Maximal pressure gradients were measured using PW Doppler and %EF and %FS were obtained from m-mode images through a short axis view of the left ventricle at the mid-papillary level. EF, ejection fraction; FS, fractional shortening, LVOT, left ventricular outflow tract; PA, pulmonary artery.
(DOCX)

S1 Fig Western blot to verify ADAMTS antibody specificity. We examined the specificity of commercial ADAMTS antibodies used to detect ADAMTS1, ADAMTS5, and ADAMTS20 by western blot. The antibody directed against ADAMTS1 used in this study was raised against a recombinant product based upon ADAMTS1 amino acids 254–725. This reagent recognizes a predominate band of approximately 80 kDa in E12.5 OFT lysate, with several weak bands at lower and higher apparent molecular masses which is consistent with reports in the literature [28–30] and the auto-proteolytic activity displayed by this isoform [29]. The antibody used to detect ADAMTS5 was prepared to recombinant product based upon the C-terminal domain of ADAMTS5, spanning amino acids 880–930. This antibody recognizes 2 major bands, at approximately 100- and 58-kDa in E12.5 OFT, which is consistent with the literature [31]. The antibody used to detect ADAMTS20 was prepared from a synthetic peptide based upon the catalytic domain of human ADAMTS20. This antibody recognizes a species of about 56-kDa, and a second species with an apparent molecular mass of greater than 200-kDa, which is consistent with the processed and full length protein respectively (http://www.phosphosite.org/proteinAction.do?id=5138862&showAllSites=true) [32].
(TIF)
