# Supplementary material for: Galnt1 Is Required for Normal Heart Valve Development and Cardiac Function
Source: PLoS One. 2015 Jan 23;10(1):e0115861. doi: 10.1371/journal.pone.0115861 (PMC4304789; doi:10.1371/journal.pone.0115861)
Supplement: S2 Table — Mice with valvular stenosis demonstrate severely elevated peak pressure gradients near the affected valves and a corresponding decline in left ventricular function. Maximal pressure gradients were measured using PW Doppler and %EF and %FS were obtained from m-mode images through a short axis view of the left ventricle at the mid-papillary level. EF, ejection fraction; FS, fractional shortening, LVOT, left ventricular outflow tract; PA, pulmonary artery. (DOCX) [file pone.0115861.s002.docx]

**Table S2.** Comparison of LV function and arterial pressure gradients in mice with aortic and/or pulmonic stenosis to mice with no valvular lesions.

|  | %EF | %FS | Max LVOT pressure gradient (mmHg) | Max PA pressure gradient (mmHg) |
| --- | --- | --- | --- | --- |
| Mice with stenotic valves. (N=17) | 43 | 21.5 | 35 | 11 |
| Mice with normal valves. (N=19) | 55 | 28 | 6 | 2 |
| p value | 0.0002 | 0.0002 | 4E-06 | 0.02 |

**Table S2**. Mice with valvular stenosis demonstrate severely elevated peak pressure gradients near the affected valves and a corresponding decline in left ventricular function. Maximal pressure gradients were measured using PW Doppler and %EF and %FS were obtained from m-mode images through a short axis view of the left ventricle at the mid-papillary level. EF, ejection fraction; FS, fractional shortening, LVOT, left ventricular outflow tract; PA, pulmonary artery.
