# Supplementary figures and images for: Galnt1 Is Required for Normal Heart Valve Development and Cardiac Function
Source: PLoS One. 2015 Jan 23;10(1):e0115861. doi: 10.1371/journal.pone.0115861 (PMC4304789; doi:10.1371/journal.pone.0115861)

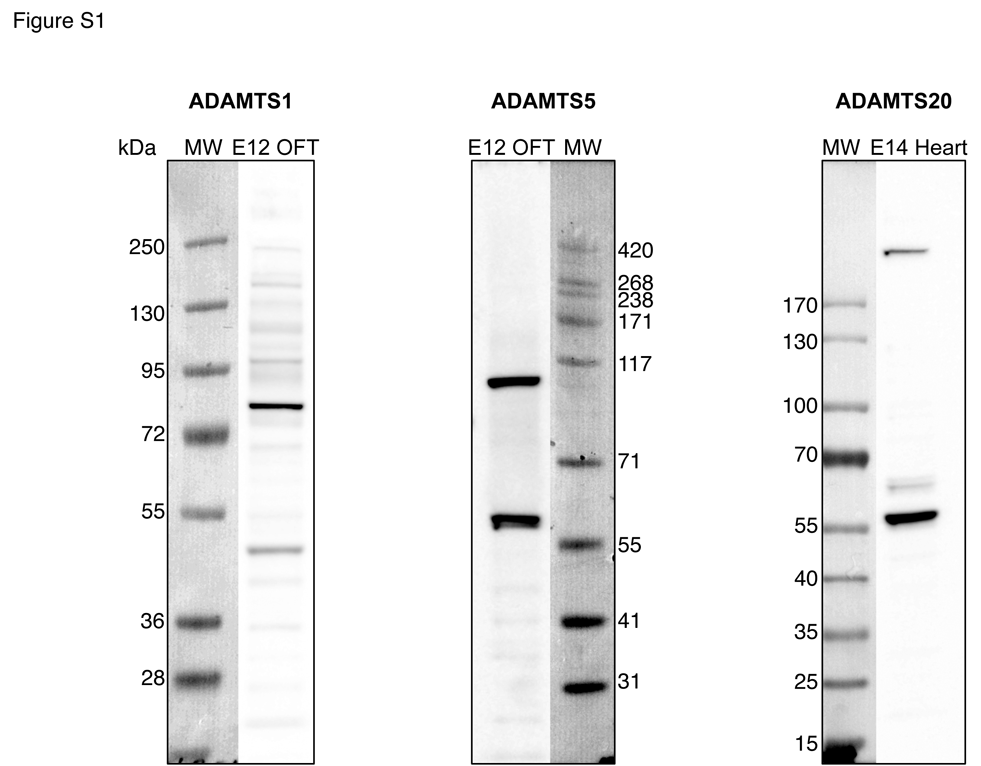

Supplement: S1 Fig — We examined the specificity of commercial ADAMTS antibodies used to detect ADAMTS1, ADAMTS5, and ADAMTS20 by western blot. The antibody directed against ADAMTS1 used in this study was raised against a recombinant product based upon ADAMTS1 amino acids 254–725. This reagent recognizes a predominate band of approximately 80 kDa in E12.5 OFT lysate, with several weak bands at lower and higher apparent molecular masses which is consistent with reports in the literature [28–30] and the auto-proteolytic activity displayed by this isoform [29]. The antibody used to detect ADAMTS5 was prepared to recombinant product based upon the C-terminal domain of ADAMTS5, spanning amino acids 880–930. This antibody recognizes 2 major bands, at approximately 100- and 58-kDa in E12.5 OFT, which is consistent with the literature [31]. The antibody used to detect ADAMTS20 was prepared from a synthetic peptide based upon the catalytic domain of human ADAMTS20. This antibody recognizes a species of about 56-kDa, and a second species with an apparent molecular mass of greater than 200-kDa, which is consistent with the processed and full length protein respectively (http://www.phosphosite.org/proteinAction.do?id=5138862&showAllSites=true) [32]. (TIF) [file pone.0115861.s003.tif]
